# Are First-Year Emergency Medicine Residents Still Behind on Level 1 Care-Based Milestones?

**DOI:** 10.7759/cureus.49842

**Published:** 2023-12-02

**Authors:** Nathan D Stuempfig, Julie Cueva, Lindsay MacConaghy, Madeleine Alexeeva, Peter Moffet

**Affiliations:** 1 Emergency Medicine, Kaiser Permanente Central Valley, Modesto, USA; 2 Emergency Medicine, Maimonides Medical Center, Brooklyn, USA; 3 Emergency Medicine, Guthrie Robert Packer Hospital, Sayre, USA; 4 Emergency Medicine, Kaweah Health Medical Center, Visalia, USA; 5 Emergency Medicine, Virginia Commonwealth University, Richmond, USA

**Keywords:** faculty evaluation, self-assessment, emergency residency, emergency medicine resident, milestones assessment and evaluation

## Abstract

Background

The Accreditation Council for Graduate Medical Education defines Level 1 as “the resident demonstrates milestones expected of an incoming resident,” yet a previous study of emergency medicine (EM) interns showed most were not meeting Level 1 milestones. In addition, previous research indicates that residents often provide more favorable self-assessments when compared to faculty assessments. Our study, performed in July 2022, aims to determine whether incoming EM residents remain behind on Level 1 care-based milestones and if resident self-assessments are consistent with faculty assessments.

Methodology

This is an observational study involving five distinct EM residency programs. Incoming interns were directly assessed by faculty for behaviors associated with the care-based milestones for EM using a standardized survey. Interns were asked to complete this same survey regarding their own performance.

Results

Faculty completed a total of 101 assessments on 49 residents. Of the 49 residents, 39 completed self-evaluations (80%). Achievement of Level 1 ranged from 25% to 82%. Residents had significantly higher self-assessments than faculty assessments on PC-1, PC-5a, and PC-6a. Faculty assessments were significantly higher than resident self-assessments on PC-6b.

Conclusions

Greater than 75% of incoming interns were able to meet Level 1 milestones in three of seven care-based milestones. However, there is a generalized trend toward overall improvement when compared to previous studies. Residents continue to demonstrate higher self-assessments than faculty in three separate care-based milestones and faculty rated residents significantly higher in one care-based milestone. This is consistent with previous studies.

## Introduction

Since the Milestones Project was implemented in 2012, emergency medicine (EM) residents have been evaluated using a progressive, numerical system of evaluation aimed at monitoring progress from a level of graduating medical student to that of an established attending physician using a scale of 1-5 [1]. These milestones were updated in 2021 and termed Milestones 2.0 [2]. As with the original version, Milestones 2.0 aims to measure observable skills within the core competencies of patient care (PC), medical knowledge (MK), interpersonal and communication (ICS), professionalism (Prof), practice-based learning and improvement (PBLI), and systems-based practice (SBP). Although there are significant differences between the original Milestones Project and Milestones 2.0, the care-based milestones for EM are largely unchanged with respect to the observable skills being measured.

The Accreditation Council for Graduate Medical Education (ACGME) defines Level 1 as “the resident demonstrates milestones expected of an incoming resident.” However, a previous study using care-based (PC 1-8) milestones revealed that fewer than 75% of entering residents achieved Level 1 for care-based milestones and a majority did not achieve Level 1 on four of the milestones when evaluated by faculty [3]. Furthermore, as has been shown in multiple previous studies, residents overestimated their abilities [4-6]. As these studies have been published, there have been numerous updates to Undergraduate Medical Education (UME) [7]. In addition, there were many disruptions to UME during the first two years of the SARS-CoV-2 (COVID-19) pandemic which impacted the traditional clinical clerkship experience for medical students [8-10]. This study was designed to provide updated information on the attainment of Level 1 competency in patient care milestones by incoming residents in the era of COVID-19 and the new milestone standards.

This study’s primary goal was to determine the proportion of incoming PGY-1 EM residents who consistently demonstrated behaviors associated with the achievement of Level 1 on eight of the ED care-based sub-competencies when evaluated by faculty. Based on previous studies [3] and recent disruptions to UME, we hypothesize that EM interns are still not consistently achieving Level 1 on the care-based milestones. The secondary goal of this study was to determine the difference between faculty assessment and resident self-assessment, as determined by the difference in proportions and logistic regression analysis.

This study was previously presented in abstract form at the Council of Residency Directors in Emergency Medicine Academic Assembly on March 22, 2023, in Las Vegas, NV.

## Materials and methods

This study was a collaborative, observational study conducted at five EM training programs throughout the country in July 2022. Each author contributed to the study conception, study design, and data interpretation. Incoming residents who were beginning their EM residency in July 2022 and who had graduated from medical school within the past 12 months were included. Residents with prior residency training or who had completed medical school more than 12 months prior were excluded. The Research Determination Committee for the Kaiser Permanente Northern California region determined that this study does not meet the regulatory definition of research involving human subjects.

**Figure 1:**
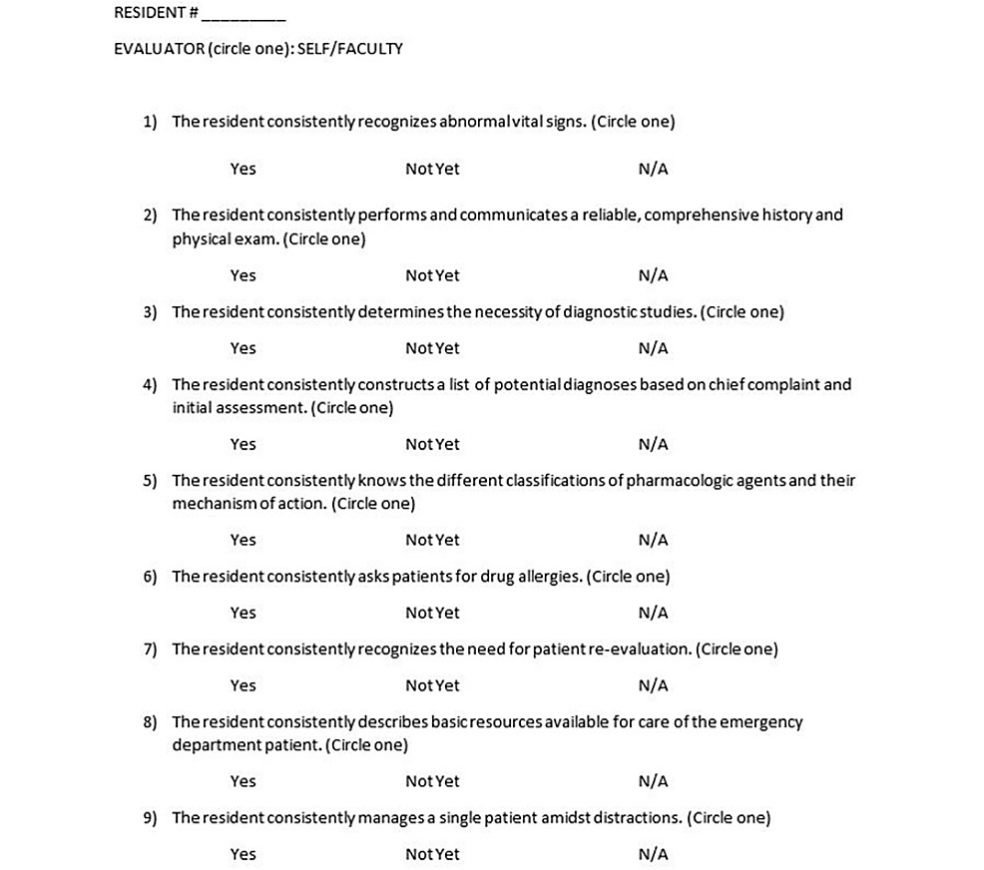
Evaluation form.

Each resident was evaluated by direct observation of a faculty member during their first several shifts in the emergency department during July 2022. A survey that has been used previously [3] was again utilized to evaluate behaviors associated with the care-based competencies (Figure 1). This survey was created by five program directors with more than 30 combined years of experience. The creators of this survey served roles on the Joint Milestones Task Force [3]. Briefly, faculty were asked to assess the resident based on nine different behaviors associated with Level 1 on eight of the emergency medicine sub-competencies/milestones. Faculty completed these evaluations at the end of the month and were asked to determine whether the residents were consistently performing these behaviors. For the purposes of the survey and assessment, “consistent” was defined as observing the behavior >75% of the time. This definition was agreed upon by the authors and utilized across all participating sites. During the same time, residents were asked to complete a self-evaluation using the same assessment. All faculty at each participating site were given personalized instruction on how to use the assessment tool before evaluating the residents. In addition, participating faculty had experience evaluating residents using traditional milestone-based assessment strategies. Residents did not receive specific training for completion of the evaluation. However, they were instructed to use >75% when referring to the term “consistently.”

Local site directors distributed evaluations to faculty and residents within their institutions at the end of July 2022. Each author, acting as a site director, compiled data which was then de-identified before being sent to the principal investigator for analysis.

Faculty member and resident reports of achievement of the nine behaviors were analyzed using proportions. Missing data on questions are reported but not considered in the analysis. For each question, the proportion reporting “yes” to the achievement of the behavior was compared between faculty and residents using Rao and Scott’s adjusted chi-square test [11]. Analysis was performed using Stata 2021 (StataCorp, College Station, TX, USA). After analysis, each author contributed to data interpretation and manuscript preparation.

## Results

Demographic data reflecting the programs and residents are presented in Table 1. The geographic delineations are those utilized by the Electronic Residency Application Service. The programs involved in this study represented three distinct geographical regions.

**Table 1:** Resident demographic data.

Residents	N = 49
Mean ± SD age	29.4 ± 3.6
Sex	Male (25)
	Female (24)
Degree	MD (35)
	DO (14)
Region of medical school attended	Northeast (4)
	Middle Atlantic (13)
	South Atlantic (10)
	East South Central (3)
	West South Central (3)
	Mountain (8)
	Pacific (4)
	International (1)
Residency program region	Middle Atlantic (2)
	South Atlantic (1)
	Pacific (2)
Residency program setting	Academic (1)
	Community (4)

There were a total of 101 faculty ratings of 49 interns across five academic medical centers as well as 39 intern self-assessments (80%). Faculty were most likely to report consistent resident behaviors in the performance of a reliable history/physical (Question 2) and the ability to manage a single patient without distractions (Question 9) (Table 2). They were least likely to demonstrate consistent behavior with understanding the classification of pharmacologic agents and their mechanisms of action (Question 5); however, this was also the area with the fewest ratings (approximately 25% without an assessment). Residents self-assessed their most consistent behaviors to be recognizing abnormal vital signs (Question 1) and recognizing the need for patient re-evaluation (Question 7). They also were least likely to report understanding the classifications of pharmacologic agents (Question 5).

**Table 2:** Faculty evaluation versus resident self-evaluation. *: Evaluators rated competency as displaying the action consistently (>75% of the time). **: Rao and Scott’s adjusted chi-square. CI: confidence interval

Milestone	Level 1 descriptor	Resident rating*	Faculty rating*	Difference	P-value**
		% (n = 39)	% (n = 101)	% (95% CI)	
PC-1	Detects abnormal vital signs	97 (38)	74 (75)	23.0 (8.4-33.0)	0.003
PC-2	Reliable history and physical	72 (28)	83 (83)	-10.4 (-26.0-7.8)	0.261
PC-3	Need for diagnostic studies	41 (16)	56 (57)	-15.4 (-35.0-2.5)	0.119
PC-4	Differential diagnosis	72 (28)	76 (77)	-4.4 (-20.0-14.0)	0.567
PC-5a	Drug classification	25 (10)	25 (25)	0.8 (-19.0-14.6)	0.920
PC-5b	Elicits drug allergies	62 (24)	43 (43)	19.0 (0.01-36.5)	0.042
PC-6a	Patient re-evaluation	90 (35)	65 (65)	25.0 (7.9-37.7)	0.006
PC-6b	Knowledge of resources	36 (14)	65 (65)	-25.5 (-45.3-8.5)	0.012
PC-7	Patient care with distraction	82 (32)	80 (80)	2.8 (-14.8-16.5)	0.709

**Figure 2:**
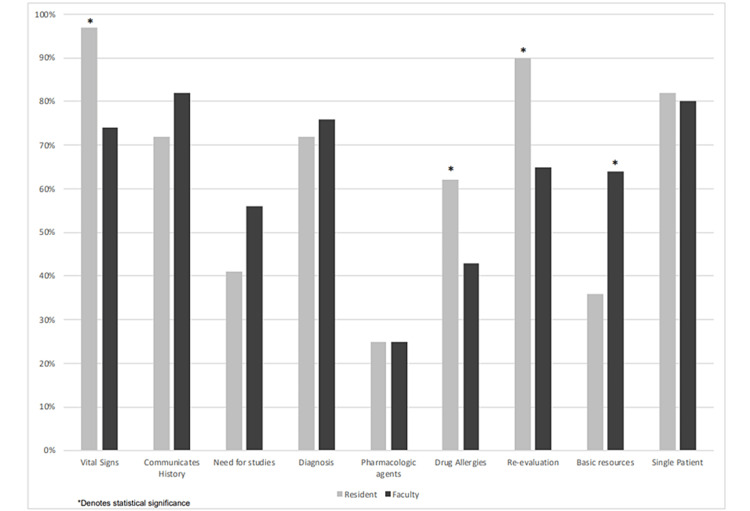
Faculty evaluations versus resident self-evaluation. *: Denotes statistical significance.

Ratings of faculty and residents differed significantly in several categories. Residents were more likely to rate themselves higher in the behaviors of recognizing abnormal vital signs (Q1, p = 0.003), asking patients for drug allergies (Q6, p = 0.042), and recognizing the need for patient re-evaluation (Q7, p = 0.006). Faculty were more likely to rate residents higher in describing basic resources available for the care of the emergency department patient (Q8, p = 0.012) (Table 2, Figure 2).

## Discussion

The primary finding of our study was that faculty were most likely to report consistent behaviors among incoming interns when required to recognize abnormal vital signs as well as their ability to manage a single patient among distractions. While faculty rated these behaviors consistently among residents, the residents themselves diverged on their own self-assessments of these behaviors. Specifically, residents were more likely to rate themselves highly in recognizing abnormal vital signs and recognizing the need for reassessment. Faculty were more likely to believe residents performed a comprehensive history, determined the necessity of diagnostics, and were able to describe the resources to care for an emergency department patient.

Our study continues to demonstrate significant differences in faculty and resident self-assessment of care-based milestones in the emergency department. The differences in the faculty and resident self-assessments are not surprising. Previous studies have demonstrated that residents’ perceptions of their own abilities vary greatly from faculty evaluations [4-6]. It does not appear that familiarity with milestone assessment, updated milestones, or COVID-19 have had much impact on this overall theme. However, our study does demonstrate a few different trends.

As mentioned previously, a similar study using the first iteration of the ACGME milestones was published in 2015 [3]. Compared to this study, we found that overall, PGY-1 residents were rated higher on most sub-competencies/milestones by faculty. Previously, fewer than 75% of PGY-1 residents achieved Level 1 on any of the sub-competencies, whereas our study showed 75% of PGY-1 residents achieved Level 1 on four of the nine sub-competencies. In addition, faculty deemed a higher percentage of PGY-1 residents to have achieved Level 1 on all sub-competencies, apart from eliciting a history of drug allergies from patients (Q6). Overall, residents continue to show deficiencies in obtaining a Level 1 on a majority of the patient care-based milestones, however. Our study also demonstrates that residents ranked themselves as less likely to have achieved Level 1 than faculty in four of the nine sub-competencies, differing from the previous study which showed that residents gave themselves higher rankings in all categories.

Several factors may have contributed to our findings. The previous study was conducted in 2015, which is when the ACGME Milestones were relatively new to faculty and residents. Having more experience and a better understanding of this evaluation system may influence both faculty and resident evaluations relating to patient care. Residents may be less likely to overestimate their own perception of patient care while faculty may be more likely to recognize the nuances of patient care that demonstrate a Level 1 on care-based milestones. In addition, as the Standard Letter of Evaluation became vital to residency applications, most aspiring EM residents began completing multiple EM rotations. More exposure to patient care in the emergency department, along with increased awareness of milestone evaluation, may lead to an improved ability to obtain the skills necessary to reach Level 1 and to evaluate themselves more critically. Regarding the residents’ ability to elicit drug allergies (Q6) specifically, faculty ratings may be lower due to an increased reliance on the electronic health record to obtain this information, which is much more ubiquitous than it was in 2015. In addition, it is doubtful that most faculty are routinely monitoring residents during the entirety of the patient encounter, often relying on resident presentations and independent patient assessment to complete their evaluations of residents. It was also noted that many faculty indicated that they were unable to assess residents’ ability to describe different classifications of pharmacological agents (Q5), with approximately 25% of responses marked as “N/A”. We suspect that many faculty are simply not asking residents to explain pharmacologic agents and their mechanism of action while on a clinical shift, instead choosing to cover this information in other educational settings.

The impact of COVID-19 disruptions on UME experiences is difficult to fully understand. Lower resident self-evaluations may have been affected by fewer overall in-person clinical experiences, creating a decrease in self-confidence related to patient care. Therefore, residents may be less likely to believe they are consistently performing the behaviors in question. While residents still rated themselves higher than faculty in a majority of the sub-competencies, there were four sub-competencies in which they rated themselves lower, though only one (Q8) was significant. This contrasts with the previous study where interns rated themselves higher than faculty on all sub-competencies [3]. The impact of COVID-19 may have affected the type of patients to which learners were exposed to while completing in-person rotations in the emergency department. Workflows and evaluation methods may have been altered to meet the demands of emergency departments during the height of the pandemic. Overall, it is difficult to establish a causational relationship between COVID-19 and this evaluation trend. Further studies addressing these questions are needed.

This study is limited by the subjectivity inherent in the faculty evaluations. Although clear guidelines were provided to faculty regarding the use of the evaluation tool, how faculty observed the interns was not standardized. Some faculty may have based their ratings on direct observation of individual interns while others may have relied on patient presentations, direct questioning, or medical record documentation. In addition, while all faculty had experience with resident supervision and with the usage of milestones, the amount of experience varied from one or two years to nearly 20 years in these realms. Furthermore, there was likely variance in the type of patients of which the interns were being assessed. Some interns may have been assessed while caring for patients with less acute and less complex case presentations while other interns may have been caring for patients requiring many complex decisions, causing them to have differing evaluations from their peers. Further studies exploring similar topics would benefit from a more standardized approach to intern evaluation.

## Conclusions

Greater than 75% of incoming interns were able to meet Level 1 milestones in three of seven care-based milestones. This demonstrates that incoming PGY-1 EM residents are still not meeting ACGME expectations in many of the care-based milestones. However, there is a generalized trend toward overall improvement when compared to previous studies, despite significant changes to the undergraduate learning environment over the past few years.

Residents demonstrated higher self-assessments than faculty in three separate care-based milestones and faculty rated residents significantly higher in one care-based milestone. There were no significant differences on the remaining three. Residents giving themselves more favorable evaluations than faculty is consistent with previous studies, though it is notable that this was not the case in four of the seven measured sub-competencies.
